# Industrializing CAR-T cell therapy: impact of automation on cost and space efficiency of manufacturing facilities

**DOI:** 10.3389/fbioe.2025.1612248

**Published:** 2026-01-06

**Authors:** Anna Louisa Weltin, Ysanne De Graaf, Amir Goudarzi, Mirko Müller, Laura Herbst, Bastian Nießing, Hubertus J. M. Vrijhoef, Robert H. Schmitt

**Affiliations:** 1 Fraunhofer Institute of Production Technology (FHG), Aachen, Germany; 2 University of Maastricht, Maastricht, Netherlands; 3 Bayer Pharmaceuticals, Wuppertal, Germany; 4 Fraunhofer Institute for Cell Therapy and Immunology, Leipzig, Germany; 5 Panaxea, Den Bosch, Netherlands; 6 Laboratory for Machine Tools and Production, RWTH Aachen, University, Aachen, Germany

**Keywords:** humans receptors, chimeric antigen, t-lymphocytes, leukemia, technology, automation

## Abstract

The A-Cell Case Study published by the “Alliance of Regenerative Medicine” illustrates how Quality-by Design can be applied to the manufacturing of Advanced Therapeutical Medicinal Products (ATMPs), using Chimeric Antigen Receptor (CAR)-T cell therapy as a ‘model’ process. However, no emphasis is given to different degrees of automation in this study. CAR-T cell therapies have been developed for various forms of leukemia, such as Acute Lymphoblastic Leukemia (ALL) or Non Hodgkin-Lymphoma (NHL). As more CAR-T cell therapies reach market approval and are being considered as first- or second line treatments, the economic efficiency and scalability of the chosen production modality become increasingly critical. Currently, academic and industrial manufacturers employ a range of approaches, from fully manual and open processing to closed and automated systems. New technologies, investments and cleanroom space requirements must be considered to assess economic and spatial efficiency in cell therapy manufacturing. This study analyses the costs and space requirements of different production modalities for autologous CAR-T cell production. The analysis shows that a higher degree of automation can reduce manufacturing costs by lowering personnel costs, cleanroom grade requirements and spatial footprint. It emphasizes the importance of maximizing cleanroom efficiency to support the scalable production of cell therapies as clinical demand grows. These results underscore the need for both industry and academia to consider automated production as a strategic approach to optimize resource use in CAR-T cell manufacturing.

## A-cell case study: the CAR-T cell process and its economic perspective

CAR-T cell therapies are increasingly being considered as second or first line treatment of leukemia, marking a shift from treatment of refractory cases to earlier stages of disease (Goyco Vera et al., 2024). This shift is expected to increase demand, especially as more CAR-T cell therapies are approved for leukemia and therapies for solid tumors progress toward commercialization (Reinhard et al., 2020). In Germany, the annual demand for CAR-T cells has quadrupled within 4 years (DRST, 2023). In Europe, there was a 27% increase from 2021 to 2022 of patients receiving CAR-T cell therapy (Passweg et al., 2024) and the manufacturers of CAR-T cell therapies such as Kymriah, Breyanzi, Tecartus, Yescarta or Carvykti reported that more than 35,685 patients had been treated as of May 2025 (Levine et al., 2024; Oribiotech Ltd, 2025). The global CAR-T cell therapy market is expected to grow at a Compound Annual Growth Rate (CAGR) of 29.8% from 2023 to 2032, driven by broader clinical acceptance and regulatory approvals for new indications. This rising demand for CAR-T cell products poses a significant challenge to the current production modalities, as production capacity must scale to meet both current and future needs (Levine et al., 2017).

The main challenge in CAR-T cell manufacturing is bridging the gap between high costs of its products and the growing demand (Hernandez et al., 2018; Cliff et al., 2023). CAR-T cell therapies are expected to dominate sales of cancer drugs for children and young adults by 2026 (Abou-el-Enein et al., 2021; Yip and Webster, 2018). However, cost of current therapies are between €200,000 and €250,000 per dose (Goff, 2024) limiting widespread application of these therapies. The main adjustable cost drivers are personnel costs, the required cleanroom infrastructure and the regulatory requirements which are time-consuming and involve high energy consumption (Ran et al., 2020). Saving on personnel and cleanroom costs can result in a significant cost advantage which opens up the possibility to industrialize CAR-T cell manufacturing. (Pimenta et al., 2021; Iglesias-López et al., 2019). Given that regulatory frameworks are only adapted slowly and to a limited extent, an alternative approach involves improving manufacturing efficiency through automation and digitalization (Hay and Cheung, 2019; Lopes et al., 2020; Nießing et al., 2021; ARM - Alliance of Regenerative Medicine, 2024). Automation allows parallelization strategies to be applied that would not be possible with manual production, either from an economic point of view due to high personnel costs or from a regulatory point of view due to the high risk of product contamination. Ran et al. and Lopes et al. provide an excellent analysis of the cost categories involved in decentralized CAR-T cell therapy manufacturing, detailing expenses related to materials, equipment, personnel, and infrastructure (Ran et al., 2020; Lopes et al., 2020). However, these studies do not account for the production area required or the degree of automation integrated. When comparing different production methods or protocols, the number of products that can be manufactured annually is highly dependent on both the available space and the efficiency of the production process impacting the cost structure.

The “A-Cell Study” outlines a case study-driven approach for applying Quality by Design (QbD) principles to chemistry, manufacturing, and controls programs in the development of cell-based therapies (ARM-Alliance of Regenerative Medicine, 2024). The case study provides an overview of the CAR-T cell therapy manufacturing process and is particularly suitable for a process and production analysis. To meet rising demand, production capacity must be expanded by improving scalability, optimizing space utilization, and reducing costs. Therefore, this study analyses the costs and space requirements associated with different production modalities of autologous CAR-T cell production based on the “A-Cell-Study” process. This analysis illustrates how costs may be decreased and capacity better utilized by parallelizing and automating the complex CAR-T cell therapy manufacturing process.

## CAR-T cell products can be produced manually, semi-automated or automated

**FIGURE 1:**
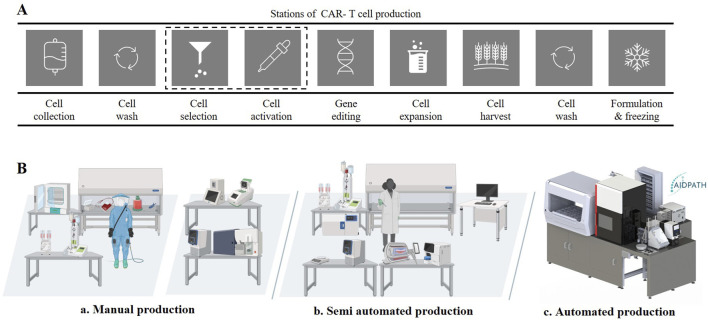
CAR-T-cells process and production modalities. **(A)** CAR-T-cell production process mapping on stations level, based on the „A-Cell Study“ **(B)** a: Manual production with high human intervention, cleanroom area and grade; b: Semi automated production with medium human interaction and medium cleanroom are and grade; c: Automated production with low human interaction, low cleanroom area and grade.

The CAR-T cell process based on the “A-Cell Study”, is summarized in Figure 1A (ARM - Alliance of Regenerative Medicine 2024). This process begins with the apheresis from which the T cells are isolated. The T cells are enriched and activated using a cell sorting method, and genetically modified with the CAR gene using either viral vectors or electroporation. The CAR-expressing T cells are expanded for a period of 3–12 days, depending on the defined protocol and use. After reaching the desired expansion level, the CAR-T cells are harvested and formulated. To ensure the safety and efficacy of the product and compliance with regulatory requirements, strict quality controls and in-process testing are carried out throughout the manufacturing process (Vormittag et al., 2018).

Three different production modalities were identified for the CAR-T manufacturing field: (a) manual production, (b) semi-automated production and (c) automated production (Figure 1B). The manual manufacturing process as described in the A-Cell Case Study, typically involves several steps that require a considerable amount of manual intervention of highly-skilled personnel (Figure 1B). In particular, seeding, transfecting and harvesting CAR-T cells are labor-intensive steps. Specialists carry out the Standard Operating procedure (SOP) of the process steps manually in suitable gowning in the corresponding GMP suites (clean room A in B) (Comission, 2022) and document the manufacturing in an analogue manner as also highlighted in the A-Cell Case study. Although this production method allows for customization and direct monitoring, it is labor-intensive and prone to variations due to human factors (Awasthi et al., 2023).

The semi-automated production modality combines both manual and automated steps, where certain unit operations are automated, while others require human intervention for customization and decision-making (Figure 1B). Many automated devices for certain unit operations are available commercially with a focus on cell expansion and washing. However, in-process control (IPCs) such as cell count, viability, immunophenotyping, etc. are typically performed manually off-line in this approach (Abou-el-Enein et al., 2021). The semi-automated approach allows for some reduction in personnel and cleanroom costs, however direct monitoring and flexibility may be limited (Aleksandrova et al., 2019; Hu et al., 2024; Culshaw et al., 2021).

Full automation of CAR-T cell manufacturing is only feasible with close-knit integration and automation of all unit operations. Some companies, such as Cellular Origins Ltd (Dr. Dan Strange, 2024) or Cellares Corporation (Grinstein, 2023) are working towards this end, as are multinational research projects such as AIDPATH (Erkens, 2022; Hort et al., 2022). In this study, the AIDPATH project will serve as an example of fully automated CAR-T cell therapy manufacturing with integrated quality control. The system integrates equipment to manufacture CAR-T cells as well as automated sampling and quality control of cell number, viability and immunophenotype (see Supplementary Figure SA1 and Supplementary). This production modality supports scalable production and high degrees of parallelization (Figure 1B).

## Economic evaluation of CAR-T cells manufacturing in manual, semi- and fully automated systems

**FIGURE 2:**
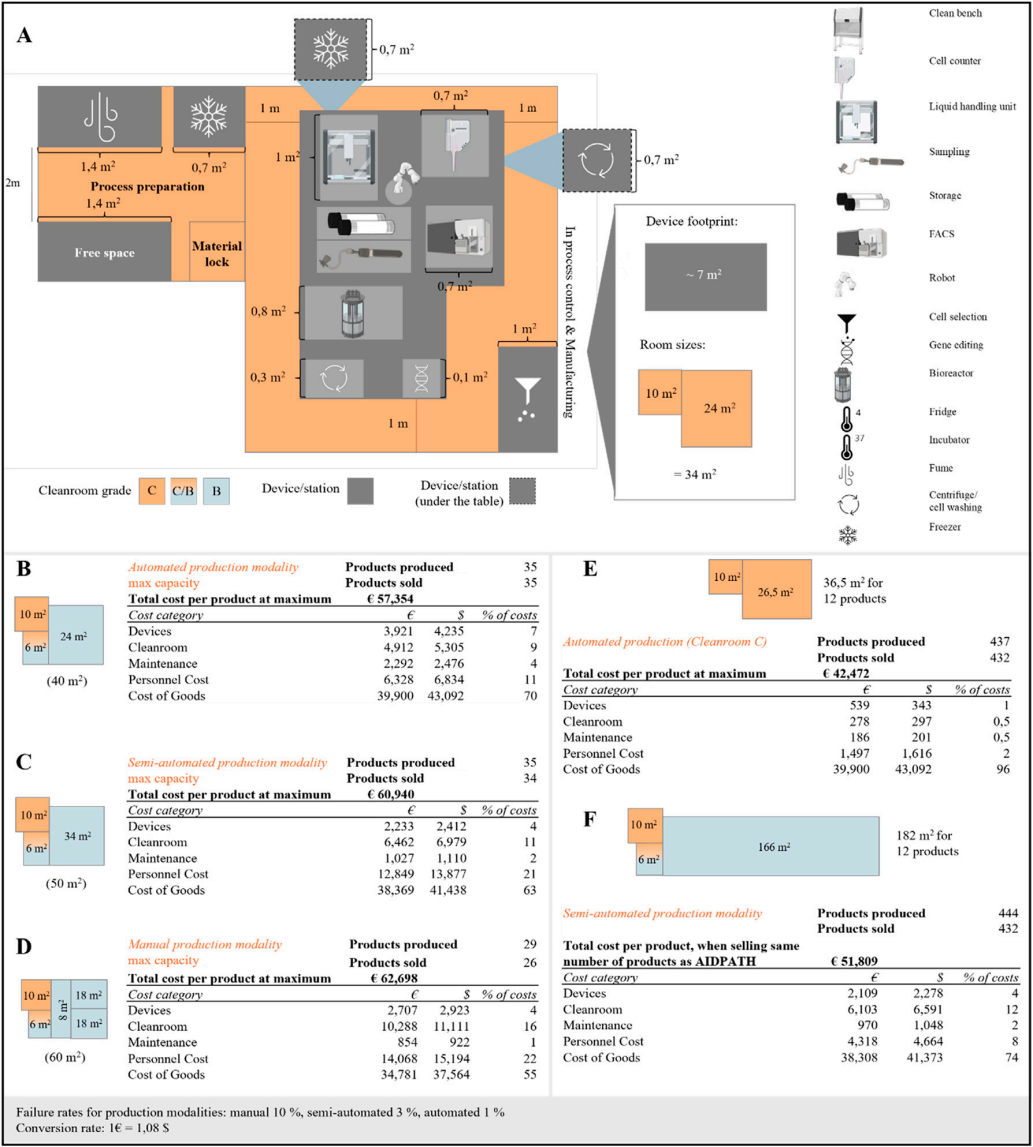
Capacity analysis and economic insights into CAR-T cell production modalities: **(A)** Capacity scenario of the automated production modality in detail, **(B)** Cost categories and total product cost of the automated production modality in a cleanroom B setting, **(C)** Cost categories and total product cost of the semi-automated production modality in a cleanroom B setting, **(D)** Cost categories and total product cost of the manual production modality in a cleanroom B setting, **(E)** Cost categories and total product cost of the automated production modality in a cleanroom C setting and scaling to 12 products in parallel, **(F)** Cost categories and total product cost of the semi-automated production modality in a cleanroom B setting and scaling to 12 products in parallel.

As the cleanroom infrastructure used in cell and gene therapy manufacturing is a cost driver of CAR-T cell therapy manufacturing, required space capacity of each modality must be estimated. This footprint of each modality is closely tied to the scalability of a given approach. For each production modality, the minimum space requirement is calculated using the device footprint and the required additional working area for any personnel involved in manufacturing, documentation or maintenance. In addition, spaces or aisles for personnel opposite the equipment must be considered to avoid obstructing activities. As the degree of automation increases, however, the needed operational space and personnel aisles decrease. These requirements result in the space allocation as described in Figure 2A for the fully automated production modality and for the other modalities in the Supplementary Figure SA3. Only the cleanrooms in the manufacturing area were considered, as these are most dependent on the respective production modality. The manual production setting requires 60 m^2^ of cleanroom space, the semi-automated production modality 50 m^2^ and the automated modality 40 m^2^ (Figures 2B–D and in Supplementary Figure SA3). For the manual and semi-automated manufacturing, this entails 47 m^2^ and 37 m^2^ of grade B cleanroom space and the remaining space being grade C cleanroom area. For the fully automated system, only 13 m^2^ grade B cleanroom area is required, illustrating the potential for cost savings when moving from manual to a fully automated production modality.

The remaining costs of CAR-T cell manufacturing were categorized into upfront, fixed yearly, and variable costs for each production modality. Upfront costs include one-time expenditures such as purchasing, transporting, and assembling manufacturing devices. Fixed yearly costs encompass equipment maintenance, cleanroom rent, staff salaries, and, where applicable, software updates. Variable costs per production primarily consist of consumables, including media, cytokines, beads, buffers, and plasticware. Cost calculations were based on electroporation-based gene editing as the reference method across all production modalities. Viral vector–based modification was not included, as its cost structure differs substantially due to the high expense of vector production and quality control requirements (Campelo et al., 2023). A detailed breakdown of manufacturing costs, including cleanroom operations, maintenance, and device-related expenses, was based on internal service cost data. Estimates for personnel time required per product were derived from experience with the production process and standard operating procedures (SOPs). For a full description of all assumptions and data sources, see Appendix (Supplementary Figure SA8).

To allow for direct comparison of the modalities, costs are expressed per product, assuming maximum annual production capacity. Maximum production capacity was calculated by dividing the number of available working days per year by the duration of manufacturing per product for each modality. In our analysis, the automated and semi-automated modalities produce 35 products per year, six more than the manual production modality at 29 products. This difference is primarily due to the longer manufacturing time required for manual production. Although variable costs remain constant per unit, fixed costs are distributed across all units produced, resulting in cost efficiencies as production volume increases.

Considering these cost categories, an economic evaluation was carried out for each production modality, with the results given in Figures 2B–D. At maximum yearly production capacity and considering the different failure rates for each modality, the cost per CAR-T treatment is approximal € 63,000 for manual production, € 61,000 for semi-automated production and € 57,000 for fully automated production. A detailed cost and resource structure per production is described in the supplementary (Supplementary Figure SA4). Across all three manufacturing modalities, the primary cost driver is the cost of goods (COG), which accounts for 55%–70% of the total cost per product (Figures 2B,C). Due to the complexity of CAR-T manufacturing, reducing reagent costs—which make up more than half of the COG—is particularly challenging. In addition to reagents, disposable materials represent a significant portion of COG. Notably, their share of total costs increases with the level of automation, as both the semi-automated and fully automated platforms rely more on highly specific, pre-packaged, single-use consumables. Another major cost driver for the automated production modality is the higher initial investment in the system itself, along with its associated maintenance costs, which are nearly double those of a semi-automated system and significantly higher than those of manual production. Interestingly, this analysis shows that using a grade C cleanroom instead of a grade B cleanroom has a minimal overall impact on manufacturing costs (see Figure 2E; Supplementary Figure SA6). However, end-to-end automation substantially reduces personnel expenses, ultimately lowering the cost per product compared to both semi-automated and manual production (Figure 2B). This cost advantage becomes evident when production volumes exceed eight products per year, as the ability to distribute fixed costs over a larger number of units plays a critical role in determining the total cost per product.

The true value of automation in the production of autologous CAR-T cell therapies lies in its ability to facilitate parallel production, which is achieved through scheduling algorithms and low failure rates. To assess the impact of parallelization on CAR-T cell manufacturing, the costs of manufacturing 12 products at the same time in a manual, semi-automated and fully automated modality are compared. The automated production scenario can treat three patients using one bioreactor, this equates to four bioreactors per platform. The cost impact of parallelization on manual manufacturing is minimal, as it primarily involves scaling out by using multiple devices operating in parallel. As a result, costs are only slightly decreased to € 61,000 per product (see Supplementary Figure SA7). However, the impact on the semi-automated and fully automated modality is substantial with costs reduced to € 52,000 (semi-automated) and € 42,000 (fully automated) per product respectively. When scaling up manufacturing, the automated production modality can produce12 products simultaneously without expanding the footprint. The investment costs for the automated production modality are distributed over the 12 CAR-T cell products, while in the semi-automated approach, operating 12 units in parallel leads to a linear increase in investment costs. The advantage of parallelization in automated production lies in reduced personnel utilization. The number of personnel hours does not increase linearly with the number of products due to the reduced need for manual interventions. The COG increases linearly for each modality, as the same input materials are required for each product due to product contact and GMP regulations (Comission, 2022). Comparison of the parallelization strategies between the automated and semi-automated modalities reveals a cost saving of approximately € 10,000 per product (Figures 2E,F). As a result, the cost of manufacturing a CAR-T cell product can be reduced by almost 20% with automated production compared to semi-automated production. For manual production, which uses open systems, parallelization can only be achieved by scaling out with devices in separate rooms. This approach minimizes the regulatory risk of contamination. The cost categories (see Supplementary Figure SA7) would therefore add up per product and increase linearly. This increased reduction of costs for increasingly automated manufacturing highlights the potential automation has over manual production at scale.

In addition to the manufacturing costs, availability of cleanroom space and scheduling of manual interventions must also be considered for scale-out of CAR-T cell manufacturing. To produce 12 products a production area of 34 m^2^ is required for the automated production modality compared to 182 m^2^ for the semi-automated production modality using the parallelization strategy broken down in the supplementary (Supplementary Figure SA5) and described in Figures 2E, F. This illustrates the scalability of the automated production modality, as five times as many products can be produced using the fully automated system when compared to the semi-automated modality in the same cleanroom space. Assuming a manufacturing duration of 10 days, over 2300 products could be produced annually, widening access of patients to CAR-T cell therapies. Overall, the parallelized automated production modality achieves 11.7 products/m^2^, whereas with the semi-automated modality about 2.4 products/per m^2^ and for the manual production modality this results in 0.5 products/m^2^ cleanroom space (Supplementary Figure SA7, cost per product). These numbers clearly highlight the importance of automation for scaled approaches in CAR-T cell manufacturing.

The failure rate can be reduced by around two-thirds in semi-automated systems compared to manual systems (Lopes et al., 2020); in automated systems, we assume a reduction of 90%, which further optimizes the economic efficiency of production. In concrete terms, this means failure rates of 10% in manual production, 3% in semi-automated production and 1% in automated production. Higher failure rates in the production modalities mean a longer production time to manufacture the same number of quality-assured products. Within 1 year, 24 more products can successfully be produced with the semi-automated production modality and 36 more in fully automated production compared to the manual production. The reduction in the failure rate yields a higher productivity for the given time and space utilized.

## Discussion and impact

Given the growing demand for CAR-T cell therapy production, this perspective highlights three areas for optimizing manufacturing efficiency and scalability: space requirements, cleanroom utilization and parallelization. These production parameters can be influenced using automation and technology enabling parallelization. However, other process-dependent cost drivers, such as COGs, are challenging to optimize without compromising product quality. The economic data in this perspective show that automated and parallelized production save the most space and costs, allowing for a cost-efficient and scalable production of CAR-T cell therapies. Reducing space requirements leads to more efficient use of a given cleanroom area and is therefore an important consideration when choosing a production modality. When automating CAR-T cells, consider the organization’s type (academic, small, large), as benefits and challenges differ (see Supplementary Figure SA9).

Considering the footprint requirements, the overall GMP-cleanroom (Comission, 2022) (Supplementary Figure SA9 in the supplementary) availability and the number of potential patients receiving cell and gene therapy treatment, another challenge can be anticipated beyond the scope of analysis in this perspective. Within this study, 10 German CAR-T cell therapy manufacturing centers were surveyed indicating a rise of 15% in patients treated from 2023 to 2024. However, this represents less than 10% of the patients eligible for CAR-T cell therapy annually in Germany now, indicating the current imbalance of manufacturing capacity to patients in need of treatment. Considering the potential approval of CAR-T cell or other Cell and Gene therapy products for, e.g., auto immune diseases (Deutsche Rheuma-Liga Bu ndesverband e. V, 2025; Klaus, 2025; Handke, 2023; National Library of Medicine, 2011) or solid tumors (Bray et al., 2024) approximately 647,000 German patients might become eligible for treatment annually. In Germany, the estimated total cleanroom space required for CAR-T cell therapy manufacturing is approximately 80,000 m^2^. With parallelized semi-automated manufacturing, it would take 3.3 years to meet the annual demand, whereas manual production would take 17 years. Only parallelized and fully automated production has the potential to meet this demand at the current cleanroom capacity in less than a year. When applying this concept to patients worldwide, at a conservative estimate, over 64 million patients worldwide could potentially benefit from CAR-T cell therapy treatment. In comparison, around 105,300 CAR-T treatments could have been administered so far (extrapolation to the world population based on the EBMT Registry). Meeting this global demand with the current standard of semi-automated production would require nearly 26.6 million m^2^ of Grade C cleanroom space, while fully automated parallelized production could reduce this requirement to 5.1 million m^2^ stressing the importance of application of automation to make therapies more widely available.

While automation and parallelisation are key technologies to increase manufacturing capacity and cost-efficiency in CAR-T cell manufacturing, emerging digital technologies might take scalability to the next level. Integrating digital twins to monitor and control critical process parameters more thoroughly and advanced process optimisation strategies using machine learning may lead to further increase in productivity and make these life-saving therapies more widely available to patients world-wide.

## Data Availability

The original contributions presented in the study are included in the article/Supplementary Material, further inquiries can be directed to the corresponding authors.
