# Supplementary material for: Industrializing CAR-T cell therapy: impact of automation on cost and space efficiency of manufacturing facilities
Source: Front Bioeng Biotechnol. 2026 Jan 6;13:1612248. doi: 10.3389/fbioe.2025.1612248 (PMC12816348; doi:10.3389/fbioe.2025.1612248)
Supplement: Supplementary file 1 [file Supplementaryfile1.docx]

**Supplementary**


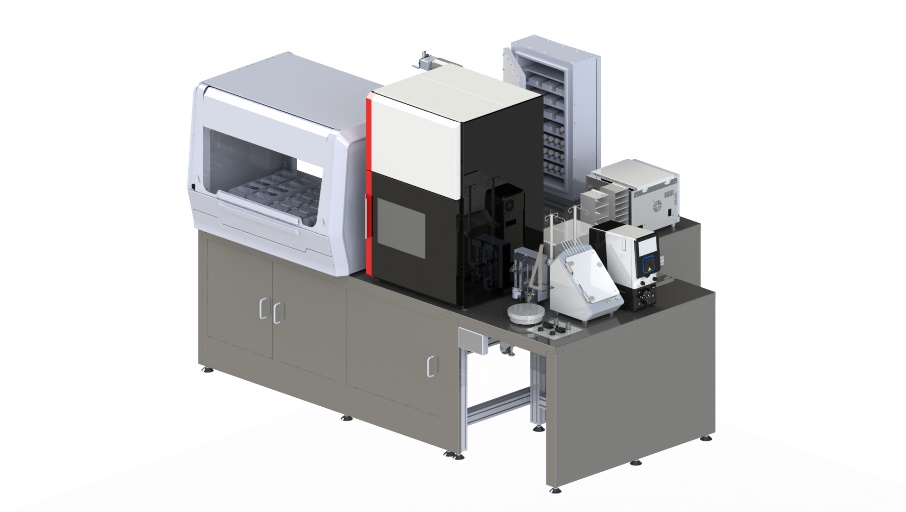


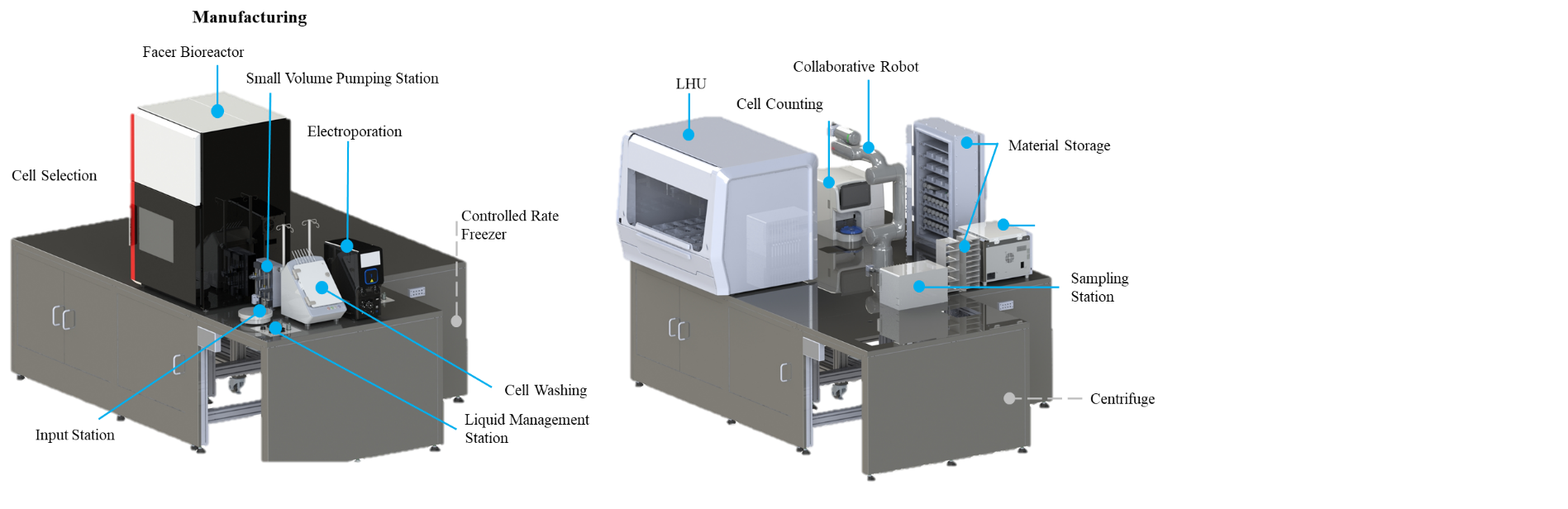


***Fig. A1: Automation-Concept for CAR-T production***, entire platform (top), bottom left (manufacturing model), bottom right (in-process control module), LHU: Liquid handling unit

**Fig. A2:** Footprint capacity of each production modality


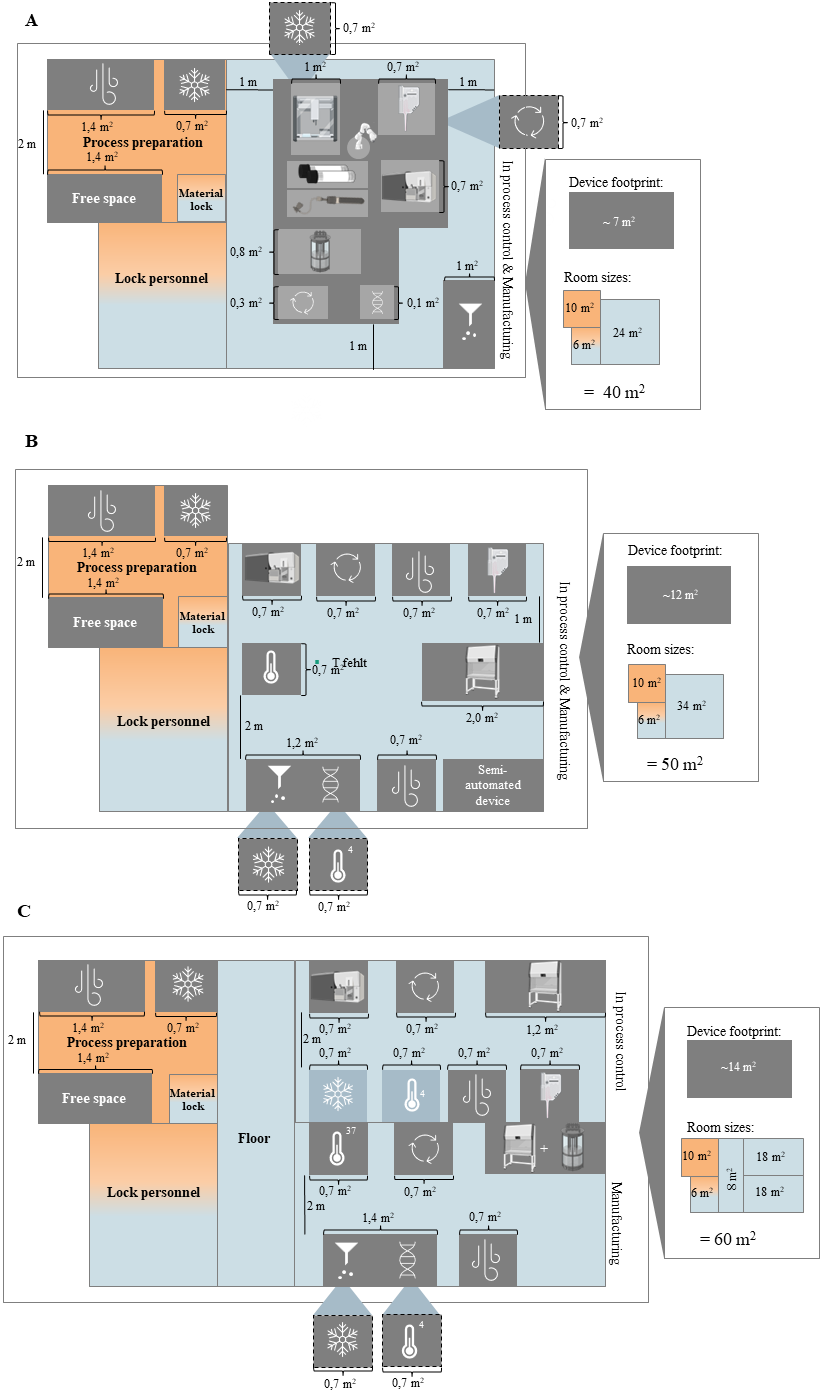


**Fig. A3:** Footprint scenarios for each production modality, **A** Manufacturing area of the automated production modality in a grade B cleanroom setting, **B** Manufacturing area needed for semi-automated production in a grade B cleanroom, **C** Manufacturing area needed for manual production in a grade B cleanroom setting.

**Fig. A4:** Cost and resource structure to estimate the CAR-T cell product cost per production modality


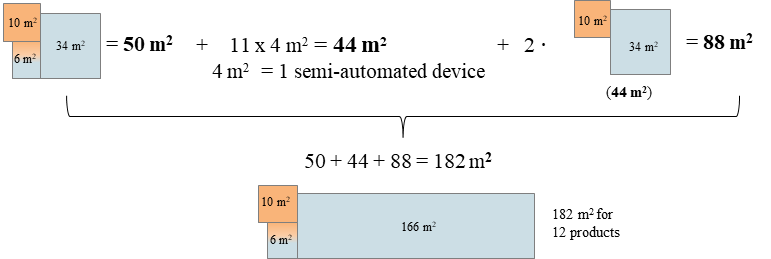


**Fig A5:** Conversative parallelization scenario for the semi-automated production modality

**Fig. A6** Automated production modality using a grade B cleanroom

**Fig. A7**: Parallelization scenario in manual production modality (12 products produced simultaneously)

| **Category** | **Assumption / Detail** | **Source** |
| --- | --- | --- |
| **Device Costs** | Cost of e.g. CliniMACS Prodigy© system, associated hardware | Internal/in-house data |
|  | Cost of AIDPATH platform (excluding VAT) | Internal procurement records |
|  | VAT exemption for intra-industry transactions (incl. maintenance services) | German tax regulation |
| **Software Costs** | COPE control software: upfront licensing + annual updates and maintenance | Internal estimates |
| **Device Maintenance** | 160 hours/year of GMP-compliant technical assistant time per modality | Internal service cost data + publicly available wage estimates |
| **Personnel Costs** | Gross salary including onboarding, social security, public sector employment benefits | Salary.com; German Employee Cost Calculator: <https://de.icalculator.com/cost-of-employee-in-germany-calculator.html> |
| **Personnel Time per Product** | AIDPATH: Simulation data and test runs | AIDPATH developers |
|  | Semi-automated: Consultant estimates based on production experience | Consultant input |
|  | Manual: Standard Operating Procedures (SOPs) | Production SOPs |
| **Facility Costs** | 1 day/year cleanroom downtime, 6-hour cleaning period between batches | Consultant input. Assumption applied to all modalities |
|  | Cleanroom operating costs/m² include materials, personnel, energy, depreciation, capital | Internal facility data |
| \| **Process Data** \| \| --- \| | Manual process | SOPs of University Hospital Würzburg, Chair of Cellular Immunotherapy and “A-cell Study” |
|  | Semi-automated process | Fraunhofer IZI, commercial CAR-T cell production |
|  | Automated process | SOPs of Fraunhofer IPT |

**Fig. A8: Assumptions and data sources for Economic Evaluation of CAR-T Cell manufacturing**

**Further explanation of Figure A8**

The total costs for the semi automated modality, associated devices, and hardware required for CAR-T cell manufacturing were estimated using in-house data. Similarly, cost estimates for the fully automated AIDPATH platform -excluding value-added tax (VAT)- are based on internal pricing data and procurement records. In Germany, intra-industry transactions, including services such as maintenance, are typically VAT-exempt, which has been taken into account in our calculations.

The cost of the COPE control software, required to operate the AIDPATH system, was calculated as a combination of one-time upfront licensing fees and annual costs for updates and maintenance over the system’s expected operational lifetime. Device maintenance costs were estimated per year, combining internal service cost data with wages for a technical assistant responsible for Good Manufacturing Practice (GMP) compliance. We assumed 160 hours of technical maintenance work per year, for each modality.

Personnel costs for manufacturing and quality control per product include gross salaries for key staff and technicians, including employer contributions such as onboarding, social security, and other public sector employment benefits (Arbeitgeberbrutto). Salary estimates were obtained from publicly available sources, including Salary.com and the German employee cost calculator (<https://de.icalculator.com/cost-of-employee-in-germany-calculator.html>). (see also Fig. A4)

The personnel time needed to manufacture one product was estimated using AIDPATH simulation data and test runs for the automated platform, while for the semi-automated process, estimates were provided by consultants based on their hands-on experience with the production process; for the manual process, standard operating procedures (SOPs) were used.

For facility-related costs, we assumed one day of cleanroom downtime per year for maintenance and a 6-hour minimal cleaning period between production batches. Cleanroom operating costs per square meter include material and personnel expenses (including maintenance), energy consumption (electricity, heating, and cooling), as well as depreciation of buildings and equipment, including capital costs.

| The regulatory framework is based on cleanroom classifications according to the EU GMP Guidelines including Annex 1.  These are the primary source in Europe—and Germany—for GMP cleanroom classification. The most recent regulatory version is EU GMP Annex 1 (2022), which details both qualification and ongoing monitoring procedures and particle limits for Classes A, B, C, and D. Compliance with these requirements is mandated by German law under the “Arzneimittelgesetz” (AMG = Medicinal Products Act) and the Ordinance on the Manufacture of Medicinal Products and Active Pharmaceutical Ingredients (AMWHV).ISO 14644-1 Standard: This international standard specifies the allowed concentration of airborne particles for each class (ISO 1–9) and underpins the technical foundation for the GMP grades. GMP Classes A through D reference ISO 5, 7, and 8 (in operation) as their technical cleanliness standards. |
| --- |

**Fig. A8:** Underlying GMP guidelines

| **Aspect** | **Small & Medium Biopharma / Academic Centers** | **Large Biopharma** |
| --- | --- | --- |
| **Role in CAR-T Development** | **Front-runners** in innovation, early-phase (pre-clinical & clinical) development, fast turnaround, high flexibility, fit-for-purpose assays/tools | **Industrialization & commercialization** leader with end-to-end capabilities |
| **Strengths** | - Fast turnaround times- High flexibility- Fit-for-purpose assays & tools- Novel concepts (especially academia)- Patient-specific (n=1) logistics expertise | - Regulatory capital- Global supply chain leverage- Robust QMS- Extensive end-to-end capabilities- Digital process integration- Investments in automation |
| **Key Challenges** | - Limited resources & funding- GxP space & maturity gaps- Dependency on CDMOs- Manual handling practices- Scalability issues- Lack of industrial manufacturing expertise (esp. academia)- Weak process robustness lifecycle | - Global corporation inefficiencies- Slower, more complex/confusing decision-making processes |
| **Impact on Later Phases** | - Regulatory compliance risks- Product supply challenges- Phase-specific struggles (process characterization & comparability) | - Less agile in early innovation phases but stronger at scale-up and commercialization |
| **Success Factor** | Early partnerships with large pharma to embed **“minimum viable process”** principles from the start | Early collaboration with small innovators to ensure smooth transition to robust, automated, scalable platforms |

**Fig. A9:** Challenges and Characteristics for clinical grade CAR-T cell manufacturing in different institutions
